# Swelling deformation and model calculation of Mg/Ca-bentonite under the influence of salt solution

**DOI:** 10.1371/journal.pone.0323021

**Published:** 2025-05-08

**Authors:** Hongming Li, Youqian Lu, Hongri Zhang

**Affiliations:** 1 Guangxi Transportation Science and Technology Group Co., Ltd, Nanning, China; 2 School of Civil Engineering, Chongqing University, Chongqing, China; 3 School of Civil Engineering, Beijing Jiaotong University, Beijing, China; 4 Department of Civil Engineering, Shanghai Jiaotong University, Shanghai, China; Tribhuvan University, NEPAL

## Abstract

The swelling characteristics and mechanisms of bentonite are significantly influenced by the chemical environment of the pore solution, making it essential to study its behavior under different pore solutions in various engineering applications. Taking magnesium-calcium-based bentonite (Mg/Ca-bentonite) as the research object, swelling tests with different concentrations of NaCl solution were carried out to study the effect of salt solution on the deformation characteristics of bentonite. The test results show that the soil expansion rate decreases from 43.7% to 37.1% with the increase of salt solution concentration. The swelling mechanism of bentonite under the influence of NaCl solution was explained from the aspects of lattice expansion theory, diffuse double layer (DDL) theory and comprehensive factors, scanning electron microscopy (SEM) and mercury intrusion porosimetry (MIP) test were introduced to further explain it from the microscopic aspect. With the increase in salt solution concentration, SEM results reveal that the soil pores become more compact and uniform. Correspondingly, the dual-peak structure observed in the MIP results transitions as the large-pore peak disappears, gradually forming a single peak within the small-pore range. Besides, the function of the swelling and time relationship curve of bentonite was fitted; the relationship model between swelling and solution concentration was established based on this, and the swelling of NaCl solution of different concentrations was simulated and verified, which provides a theoretical model basis for the practical application and numerical simulation of bentonite engineering.

## Introduction

At present, bentonite is widely used in civil engineering, not only involving daily projects such as foundations and slopes, but also involving special projects such as tunnel excavation, underground disposal of nuclear waste, landfills, etc., in which bentonite is subject to complex environments [1–5]. The results also show that in addition to the initial dry density, water content, stress conditions and temperature, the chemical environment of the pore solution is another important factor to evaluate the swelling characteristics of compacted bentonite [6–8]. Therefore, it is necessary to study the swelling characteristics and mechanism of bentonite under different pore solutions.

In the current study on the swelling characteristics of bentonite by pore solution, smectite undergoes crystalline swelling after erosion by NaCl solution, and it was believed that the expansion, or crystalline swelling, was caused by the hydration of exchangeable cations in the crystalline interlayer, and the converse is the hydration of the particle surface [8–11]. Tripathy et al. [12] proposed a new double layer theoretical model by performing swelling tests on three different types of bentonite (MX80, Febex, and Montigel), in view of the fact that the theoretical value of the swelling force at low dry density by the Gouy-Chapman DDL theory does not match the actual value. The swelling force of clay in the soil-water system is mainly caused by the tendency of the liquid phase in the clay to diffuse into the soil-water system. The thickness of the double layer is mainly controlled by the characteristics of the pore solution, and the increase of the dielectric constant of the pore solution can lead to an increase in the thickness of the double layer, and vice versa [13–16].

Saiyouri et al. [17] believed that the number of wafer layers after the swelling of sodium-based and calcium-based bentonite was not the same, and the swelling size was also different. The interlayer resistance ions of bentonite control the composition of the microscopic composition, which directly affects the macroscopic physical and mechanical properties of bentonite, and the swelling force of calcium-based soil is higher than that of sodium-based soil, while the swelling is the opposite [18–20]. Segad et al. [21] used Monte Carlo simulation to predict that the swelling of monovalent exchange cations was larger than that of divalent cations, and the external specific surface area (BET method) of calcium-based soil was higher than that of sodium-based soil, while the internal specific surface area (EGME method) was the opposite. In summary, the difference in mineral composition of bentonite can cause bentonite to exhibit different swelling characteristics. It is not clear to use lattice expansion theory or double layer theory to explain the swelling characteristics, and even the opposite conclusion appears. It is necessary to further clarify the swelling mechanism of bentonite.

At the same time, the change of microstructure under the influence of salt solution also affects the swelling characteristics of bentonite. Through scanning electron microscopy (SEM) observation of the clay particles of smectite, illite and kaolinite under salt solution conditions, the experimental results show that the clay particles of illite are mainly dispersed salt flocculation structures, kaolinite is mainly characterized by a stable flocculation structure, and calcium-based smectite is mainly flocculation structure caused by attraction [22,23]. When the concentration of the pore aqueous solution is low, the clay particle arrangement of Na-based smectite is mainly as follows: the positively charged edge of the clay particle and the negatively charged plane attract each other, forming an edge-surface structure. When the concentration of pore aqueous solution is low, the arrangement of clay particles of sodium-based smectite is mainly as follows: the positive-edge of the clay particle and the negative-face attract each other, forming an edge-surface interaction structure [24]. The particle contact type of bentonite depends on the concentration of the pore solution. The main arrangement of clay particles of bentonite in deionized water is edge-to-face (E-F) contact. As the ion concentration increases, it gradually transforms into edge-to-edge(E-E) contact and finally becomes face-to-face (F-F) contact [25,26]. Therefore, existing research lacks systematic analysis on how changes in salt solution concentration drive the evolution of soil microstructure and how this evolution specifically affects the macroscopic deformation characteristics of the soil. On the other hand, under the influence of salt solutions, the mineral composition of expansive soils and the chemical properties of the pore solution, such as pH, undergo changes at the microscopic level. These changes affect the structural configuration between soil particles, thereby altering the physico-chemical properties of the soil [27,28]. The impact of soil-water interactions on the arrangement of soil particles remains unclear, necessitating further clarification of how microstructural evolution under the influence of salt solutions affects the macroscopic deformation of the soil.

In nature, calcium-based bentonite is abundant, while sodium-based bentonite is not. Sodium-based bentonite is generally obtained by modifying calcium-based bentonite through sodium modification [22,29]. Although there has been progress in the study of sodium-based bentonite and calcium-based bentonite under the influence of salt solutions, the research on calcium-based bentonite remains relatively limited, particularly concerning the microstructural evolution and its impact on macro deformation characteristics. Therefore, further research on calcium-based bentonite under salt solution influence is needed to better understand its behavior and properties in engineering applications. Therefore, this paper selects Ningming bentonite from Guangxi Province, China as the research object which belongs to Mg/Ca-bentonite, and carries out the expansion rate test under different concentrations of NaCl solution, studies the influence of NaCl solution on the deformation characteristics of Mg/Ca-bentonite, and analyzes the swelling mechanism of bentonite under water-soil action from the microstructure aspect. On the other hand, the main parameter variables of the current expansion model are the changes of initial water content, initial dry density and overlying load, so it is necessary to establish a relationship model between swelling deformation and solution concentration. In this paper, based on the electric double layer theory, the relationship model between swelling deformation and solution concentration is established, and the swelling deformation of calcium bentonite with different concentrations of NaCl solution is simulated and verified, which provides a theoretical model basis for the practical application and numerical simulation of bentonite engineering.

## Materials and methods

### Basic physical properties and test scheme of bentonite

**Fig 1:**
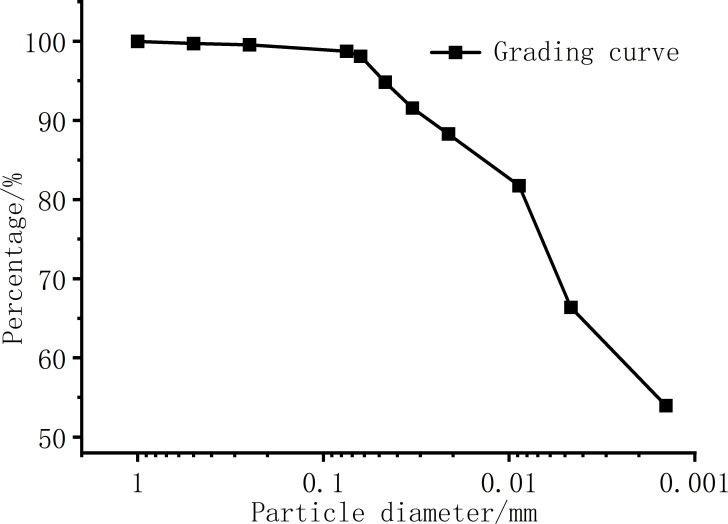
Grading curve of the Ningming Bentonite.

The test soil sample was taken from Ningming County, Chongzuo City, Guangxi Province, China, so it was named Ningming Bentonite. Fig 1 is the grading curve of the Ningming Bentonite, the proportion of clay components less than 0.005mm in bentonite exceeds 66.48%, and the proportion of silty soil of 0.075 ~ 0.005mm is 32.55%, so the bentonite is mainly composed of clay and silt. As shown in Table 1, bentonite contains a high liquid limit of 71.45%, a plasticity index of 42.80, and a high specific surface area. The type of bentonite is determined according to the content of cations. It is known from Table 1 that the content of calcium ions and magnesium ions is significantly higher than that of sodium ions and potassium ions. Therefore, Ningming bentonite belongs to Mg/Ca-bentonite. Referring to the former Soviet Union ‘s method of determining the type of bentonite by the percentage of exchangeable cations in montmorillonite to the total ion exchange amount, it is known from Table 1 that the content of calcium ion and magnesium ion is significantly higher than that of sodium ion and potassium ion, and the proportion is 50.41% and 40.50% respectively, so the soil used in this experiment belongs to Mg/Ca-bentonite. The clay mineral composition of Ningming bentonite is shown in Table 2, the main clay mineral composition is magnesium-calcium-based illite mixed layer, mixed with a small amount of kaolin and illite, and the free expansion rate is 63.5%.

**Table 1:** Physico-chemical property of Ningming Bentonite.

Soil type	Plastic limit w_p_/%	Liquid limit w_l_/%	Plasticity Index I_p_/%	Proportion G_s_	Free expansion rate(%)	Optimal moisture content(%)	Specific surface area (m^2^/g)	
Bentonite	27.65	68.32	40.67	2.74	63.5	20.1	178.2	
	Exchange volume (meq/100g)	Exchange ion composition				Total exchangeble base (meq/100g)	Proportion of Mg^2+^(%)	Salt base saturation (%)
		Ca^2+^	Mg^2+^	Na^+^	K^+^			
	19.92	3.92	4.88	0.36	0.52	9.68	50.41	49.34

**Table 2:** Fully chemistry analysis of silicate.

Soil type	Clay mineral content(%)					Mixed layer ratio(%S)	
Bentonite	S	I/S	It	Kao	C	I/S	C/S
	–	73	8	15	4	60	–

Note: S is smectite; It is illite; Kao is kaolinite; C is chlorite; I/S is illite-montmorillonite mixed-layer clay; C/S is chlorite-smectite mixed-layer clay.

### Test plan

(1) Expansion rate experiment: The effect of different concentrations of NaCl solution on the expansion rate of Ningming bentonite was tested, and the dried soil sample with an initial moisture content of 0 was pressed by static compaction method to a cutting ring sample with a dry density of 1.59 g/cm^3^. The sample was loaded into the WJ-2 soil dilatometer, and then the salt solution was added to carry out the expansion rate test. The expansion rate test was carried out with salt solutions with concentrations of 0, 0.01, 0.05, 0.1, 0.5, 1.0, and 2.0 mol/L, respectively. The criterion for judging the equilibrium of the expansion rate is that when the deformation of the expansive soil sample does not exceed 0.01 mm within 2 hours. The calculation method of the expansion rate is shown in equation (1). The influence of salt solution on bentonite was characterized by expansion rate experiments under different concentrations of NaCl solution.


$$\delta_{t}=\frac{R_{t}-R_{0}}{h_{0}}$$ (1)

In the formula, $\delta_{t}$ is the expansion rate at time t, %. $R_{0}$ is the initial value of the dial gauge, mm. $h_{0}$ is the initial height of the sample, mm.

(2) MIP and SEM test: The same ring specimens with a water content of zero were placed in a stacked saturator and saturated with salt solutions of corresponding concentrations for 24 hours. After saturation, the specimens were removed, cut into small pieces, frozen in liquid nitrogen at -196°C, and dried under a vacuum environment at -63.5°C for 24 hours. The dried specimens were then subjected to MIP and SEM analyses.

## Results

### Expansion test

**Fig 2:**
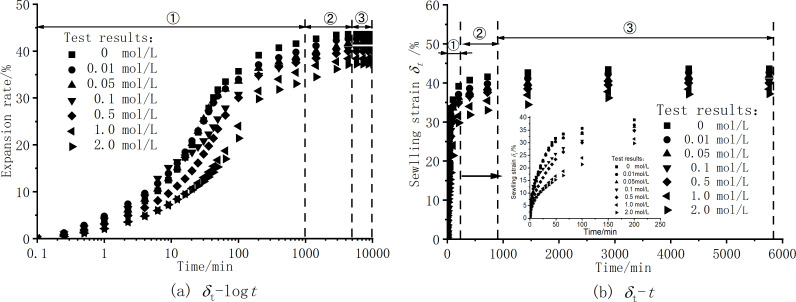
Variation curves of sample expansion rate and time under different concentration solution conditions. (a) $\delta_{t}-logt$ , (b) $\delta_{t}-t$.

Fig 2 shows the expansion rate curve of the sample over time under different solution concentrations. Fig 2a illustrates the *δ*_*t*_-log*t* swelling curve, the swelling of the soil reaches equilibrium after 6000 minutes. Therefore, establishing 6000 minutes as the endpoint for the *δ*_*t*_-*t* swelling curve. The expansion rate-time curve is a typical three-stage swelling process: ① fast stage, ② deceleration stage, ③ slow stage [30–32]. After the dilatometer is injected with the solution, the expansion rate within 0 ~ 120 mins rises rapidly, almost linearly, and the water quickly enters the pores and reacts with the hydrophilic clay minerals of the soil, so that the thickness of the DDL on the surface of the clay mineral particles increases. Therefore, as the soil continuously absorbs water, the thickness of the DDL on the surface of the soil particles increases continuously, and the growth rate of the expansion rate begins to decrease and enters the deceleration swelling stage of 120–720 mins. The strain curve increases linearly, which indicates that the rapid phase of the initial swelling develops relatively quickly, possibly because the initial swelling depends primarily on the rate of dissipation of the base suction rather than the chemical composition of the invading solution. However, as shown in Fig 2, during the invasion of NaCl solution, as the concentration of the salt solution increases, the time for the swelling strain to stabilize becomes longer, and the swelling development in the deceleration phase is slower. This phenomenon may be caused by the diffusion of salts and cation exchange, which prolong the process of swelling strain to a steady state. With the continuous invasion of the solution, the moisture content of the sample continues to increase, and the soil finally enters the third stage, that is, the slow swelling stage, until the expansion rate finally tends to stabilize.

**Fig 3:**
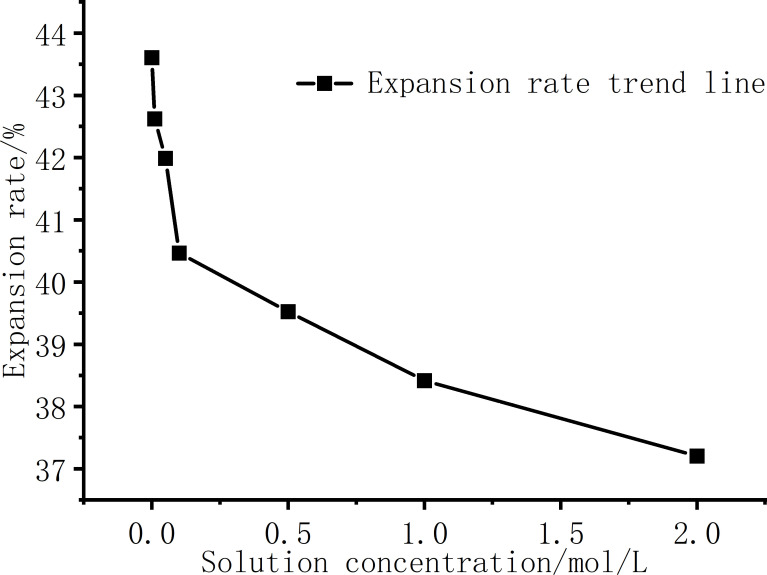
Relationship between expansion rate and NaCl solution concentrations.

Fig 3 presents the expansion rate curves under different NaCl solution conditions. As shown in Figs 2 and 3, with the increase of NaCl solution concentration,the expansion rate of the sample decreases gradually, and the decreasing trend of the expansion rate is obvious when the concentration is low. As the concentration increases, the expansion rate does not decrease all the time, but the decreasing trend is gradually gentle. The expansion rate of pure distilled water without salt is 43.61%, and the expansion rate of c = 2.0mol/L salt solution sample is 37.21%. The NaCl solution inhibits the swelling of bentonite and has an obvious inhibitory effect. Basically, the influence of the initial structure formed by the initial moisture content during sample preparation can be ignored, and the concentration of the solution inside and outside the sample can be balanced when different concentrations of salt solution invade, so the sample has a high expansion rate.

### SEM and MIP

**Fig 4:**
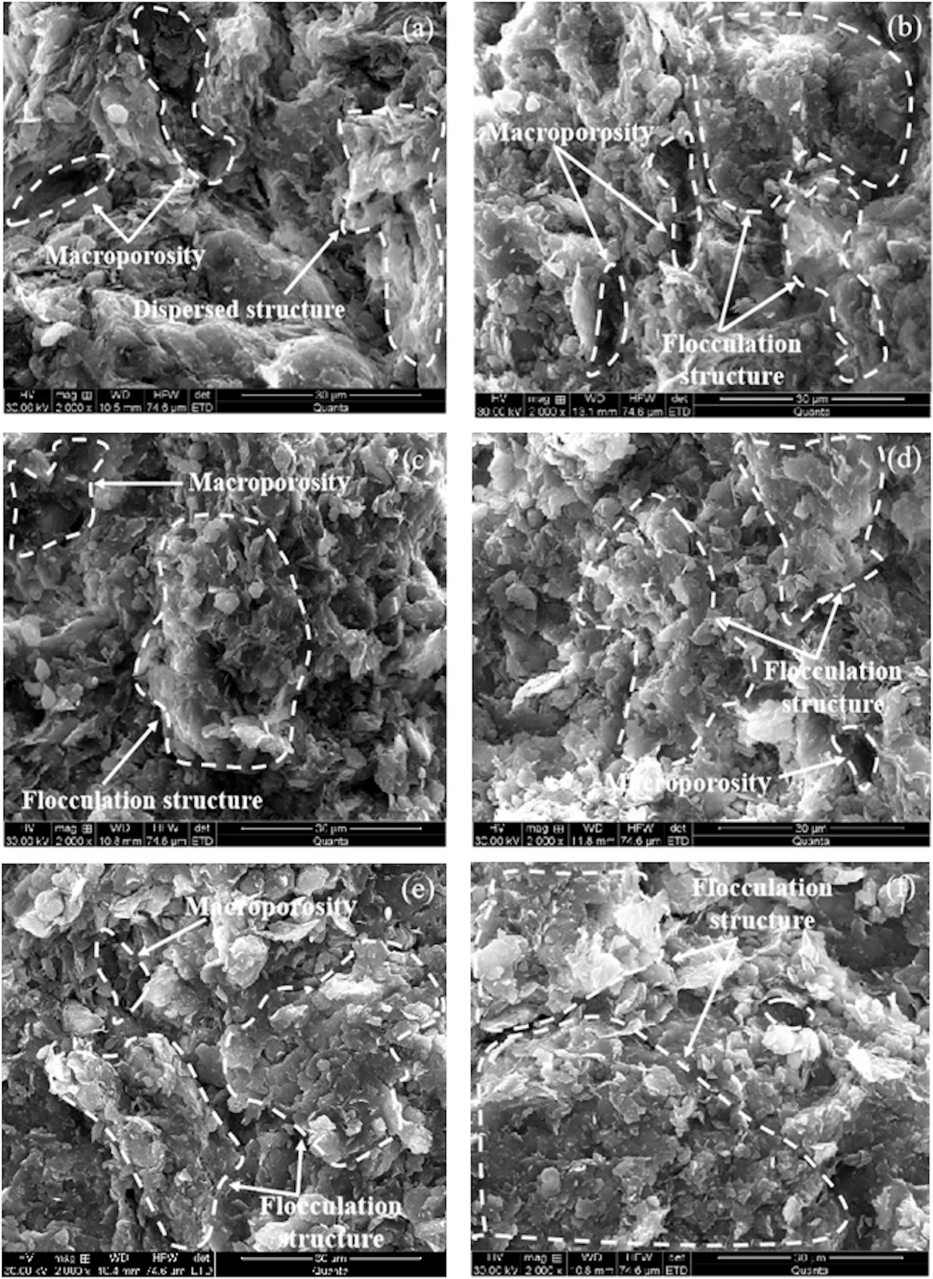
SEM test results of soil under different NaCl solution concentrations : (a) c = 0 mol/L, (b) c = 0.01 mol/L, (c) c = 0.1 mol/L, (d) c = 0.5 mol/L, (e) c = 1.0 mol/L, (f) c = 2.0 mol/L.

The results of SEM under varying concentrations of salt solution are shown in Fig 4, the SEM experimental results indicate that the samples saturated with pure water exhibit a relatively loose and irregular particle arrangement, with numerous intersecting structures and varying pore sizes between particles. When the saturating solution is replaced with NaCl solution and the concentration gradually increases, the clay particles within the soil samples transition from mixed-type contacts to predominantly face-to-face contacts. This change alters the arrangement sequence of the clay particles, resulting in a more orderly and closely packed parallel flocculation. At 2000x magnification, this transformation is clearly visible, as the originally interlaced particles shift to parallel or near-parallel alignments, with sheet-like particles attracting each other and flocculating to form aggregates. These aggregates then further bind into larger clusters through the interaction between cementing substances and the aggregates. As the concentration of the saturating solution increases, face-to-face contact becomes the dominant contact type between particles, leading to a more compact and uniform particle arrangement within the soil. The content of large and medium-sized pores gradually decreases, with medium and small pores becoming the predominant type of interparticle voids.

**Fig 5:**
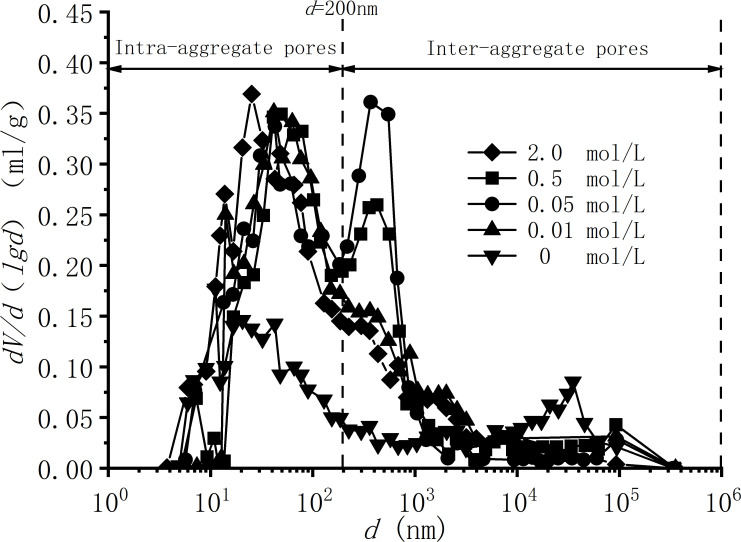
Relationship curve between pore size and pore size distribution density.

Fig 5 is the pore size and pore size distribution density curves obtained from the MIP experiments of samples under different concentrations of NaCl solutions. The mercury intrusion curves have an obvious bimodal structure, and the pore sizes are mainly distributed between 4 ~ 4.0x10^5^nm. According to the research of Ma et al. [33], *d* = 500 nm can be used as the dividing line between the inside and outside of the pore aggregates of Ningming bentonite soil. In this experiment, *d* = 200nm was selected as the threshold, which differs from the research results [33]. This difference arises because the influence of salt solutions induces flocculation in the soil, leading to a more uniform pore structure and a smaller threshold value for the boundary. As the NaCl concentration increases, with *d* = 200 nm as the dividing line in this experiment, the large pores on the right gradually decrease, and the small pores on the left continue to increase. At the same time, the results of the MIP test is consistent with those of SEM tests. As shown in Fig 5, the sample of pure aqueous solution has a peak in the range of 1.0x10^4^ ~ 1.0x10^5^nm, that is, the sample has large pores and less small pores, which is consistent with the results of large pores in the SEM test results in Fig 4a. With the increase of solution concentration, the content of macropores in the MIP test results gradually decreases, the content of small pores gradually increases, and the range of pore size gradually migrates to small pores, which is consistent with the SEM test results that the macropores of the sample become less and gradually become uniform and dense.

## Discussion

When studying the influence of invasive solutions on the swelling of bentonite, it is particularly important to explore the swelling and contraction mechanism of bentonite. The swelling of the sample is generally due to the inclusion of smectite, illite and other expansive clays. At present, the most important swelling theories are lattice expansion theory and the DDL theory, the following will investigate bentonite swelling considering the influence of these two aspects, particle contact mode and other comprehensive effects on salt solution analysis and discussion.

### Lattice expansion theory

**Fig 6:**
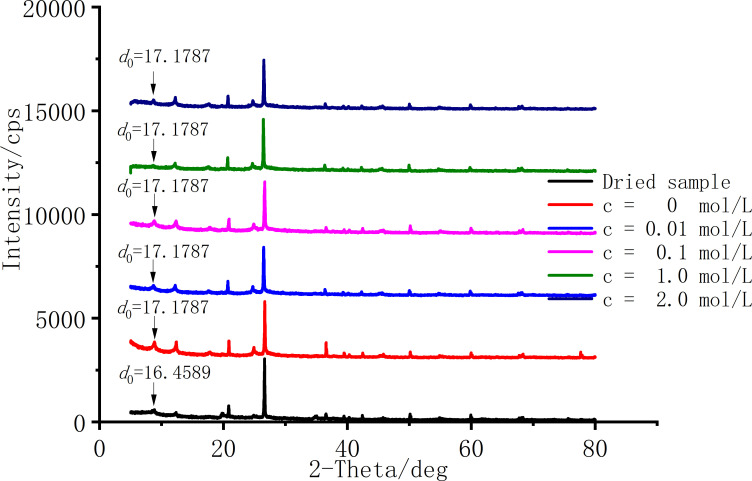
Interlayer spacing results at different saline concentrations.


Clay minerals form crystalline interlayers through two basic structural units of aluminum-oxygen octahedral (O) and silicon-oxygen tetrahedron (T), and different binding ratios lead to differences in clay swelling properties. Smectite belongs to 2:1 type (TOT type) clay mineral, the connection force of OH^-^ and O^2-^ between the basic structural units of the crystalline interlayer is weak, and water molecules can enter the layer to form a hydration film to cause the crystalline interlayer to expand, resulting in violent water absorption swelling. After the expansion rate test, the sample was disassembled for X-ray diffraction (XRD) test, and the incidence angle (2*θ*) selected for the experiment was a high angle range of 5° ~ 80°, and the low angle of 1° ~ 5° was found to have no peak after the test, so it was not scanned. Fig 6 shows the interlayer spacing results under different salt solution concentrations. The results show that the diffraction angle (*θ*) of smectite is about 8.738°, and the interlayer spacing of the dried sample is 16.4589Å. Whether it is pure aqueous solution or salt solution, the interlayer spacing of smectite is 17.1787Å, and the interlayer spacing does not change with the increase of solution concentration.

$$d=\frac{\lambda }{2\sin\theta }$$ (2)

In the formula, d is the interlayer spacing, nm;λ is the X-ray wavelength, which is 0.154056nm;θ is the diffraction angle.

The main mineral component of Ningming bentonite is the mixed layer of illite and smectite, which accounts for 73%. Illite and kaolin, which account for a small proportion, does not expand the crystalline interlayer due to the characteristics of mineral crystal structure. Bentonite with crystalline swelling will generally show swelling several times the size of the sample [17–21], but Ningming soil bentonite does not undergo severe swelling when it absorbs water and expands, and XRD test results show that smectite does not undergo crystalline swelling, so it can be judged that its illite and smectite mixed layer is more manifested as the properties of illiite. On the other hand, the above results also correspond to Table 1 that Ningming bentonite belongs to Mg/Ca-bentonite; compared with nano-based bentonite, because calcium and magnesium ions have stronger van der Waals forces than sodium ions between crystalline interlayers, water molecules are relatively more difficult to enter between crystalline interlayers, and the corresponding Mg/Ca-bentonite bentonite swelling is weaker [20,21]. Therefore, the swelling of the crystalline interlayer of Ningming bentonite is not obvious when it swells after absorbing water, and can basically be ignored.

### Electric double layer theory

The high-valence silicon-aluminum ions in O and T, the basic structural units of clay, are replaced by low-valence calcium and magnesium ions due to isomorphic replacement, resulting in negative charges on the surface of clay particles. Negative charges adsorb cations in water, making the cations align on the surface of clay particles to form the DDL, and the cations adsorb water molecules to form a hydration film. The thickness of the hydration film is the thickness of the DDL. The greater the thickness of the double layer, the larger the distance between clay particles, and the macroscopic performance is that the swelling deformation of clay is greater when it absorbs water. The thickness of the DDL on the surface of clay particles can be calculated according to the following formula (3).


$$H={\left(\frac{\varepsilon_{0}DkT}{2n_{0}e^{2}v^{2}}\right)}^{1/2}$$ (3)

Where *H* is the thickness of the double layer; $\varepsilon_{0}$ is the vacuum coefficient, $\varepsilon_{0}=8.8542\times 10^{-12}C^{2}J^{-1}m^{-1}$; *D* is the dielectric constant, F/m; *k* is the Polzmann constant, $k=1.38\times 10^{-16}ergsK^{-1}$; *K* is the thermodynamic temperature; $n_{0}$ is the solution concentration; *e* is the unit charge. v is the cationic valence.

**Fig 7:**
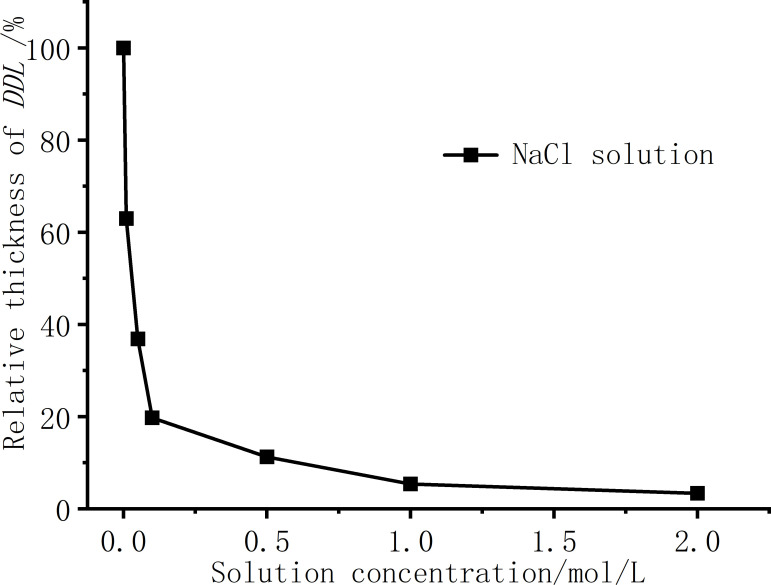
The relationship between the relative thickness of the double layer and the NaCl concentration.

Table 3 is the permittivity of different concentrations of solution, and the relative thickness of the DDL with the concentration of NaCl solution is obtained by bringing the dielectric constant into equation (3), as shown in Fig 7.

**Table 3:** The dielectric constant *D* of the solution.

concentration/mol/L	Dielectric constant/F/m
0	80
0.01	79.9
0.1	78.8
0.5	74.1
1.0	69.0
2.0	60.6

**Fig 8:**
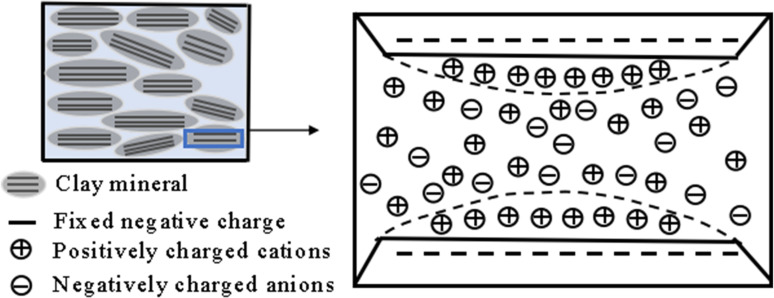
Structure diagram of diffusion electric double layer.

The schematic diagram of the DDL structure between two platy soil particles is shown in Fig 8. The soil particles themselves carry fixed negative charges. The Stern layer, also known as the adsorption layer, consists of cations strongly adsorbed near the soil particles due to intense electrostatic forces. The diffuse layer extends from the edge of the adsorption layer to the bulk solution and is composed of hydrated ions and cations, with an uneven distribution. Closer to the soil particles, the ion concentration is higher. The thickness of the DDL influences the swelling rate of the soil. As shown in Fig 8, when the solution is in the low concentration range, the relative thickness of the DDL drops sharply with the emergence of ions, and when the concentration is greater than c = 0.5 mol/L, the thickness of the DDL slowly decreases. The relative thickness of the DDL rapidly decreases from 100% to 11.26%, corresponding to a reduction in the soil swelling rate from 43.7% to 39.3%. The relative decrease in the swelling rate reaches 70.1%. That is, the change of the double layer thickness is more sensitive at low concentrations. On the other hand, the salt solution will inhibit the thickness of the DDL, Na^+^ will first replace the Ca^2+^, Mg^2+^, Al^3+^ plasma in the bentonite crystalline interlayer structure, the higher the concentration of Na^+^, the more high-valent cations are displaced, and the replaced cations contain higher valence bonds than Na^+^ [18]. The increase in the concentration of high-valence cations and Na^+^ ions will not only enhance the hydration process of cations, but also compress the thickness of the DDL. The increase in the concentration of high-valent cations and Na^+^ ions [5,34], in addition to enhancing the hydration process of cations to compete for water molecules in the DDL, thereby inhibiting the expansion rate of the soil, will also compress the thickness of the DDL. The macroscopic manifestation shows that with the increase of NaCl solution concentration, the expansion rate of the soil decreases gradually, and the final expansion rate also decreases. The trend of the relationship curve between the relative thickness of the double layer and the NaCl concentration is generally consistent with that of the expansion rate versus NaCl concentration in Fig 3, which indicates that the swelling of bentonite used in this experiment is mainly affected by the DDL, so the NaCl solution has a significant inhibitory effect on the swelling of bentonite.

### Comprehensive effect

The swelling deformation of bentonite can sometimes be explained by lattice expansion theory or electric double layer theory according to different soil types, and in some cases it is the result of the mutual coupling of the two or the combined action of other factors, that is, the swelling and contraction characteristics of bentonite are the result of the comprehensive physical-chemical action of clay in water-soil. In particular, the system of ion and soil composition of bentonite under the erosion of salt solution, that is, in the water-soil-electrolyte system, the electrification of the clay surface and the resulting osmotic pressure will play a decisive role in the swelling and contraction characteristics of the soil, and the corresponding soil hydration process will be macroscopically manifested as the swelling and contraction process of the soil, that is, surface hydration and osmotic hydration. On the other hand, the influence of the contact mode of clay sheet structural units on the swelling and contraction characteristics of soil in water-soil interaction cannot be ignored. Different interactions lead to different structures, which ultimately affects the swelling and contraction characteristics and strength characteristics of the soil, while the F-F interaction unit presents the smallest relative height among the three contact types, that is, the soil has the lowest expansion rate. Different soil mineral components and solution pH values would show different contact modes of structural units [18,22]. On the other hand, soil particles in salt solutions often undergo intense water-soil interactions, affecting the pH of the solution and subsequently altering the arrangement of soil particles [18,27]. This aspect of mechanistic research is often overlooked.

**Fig 9:**
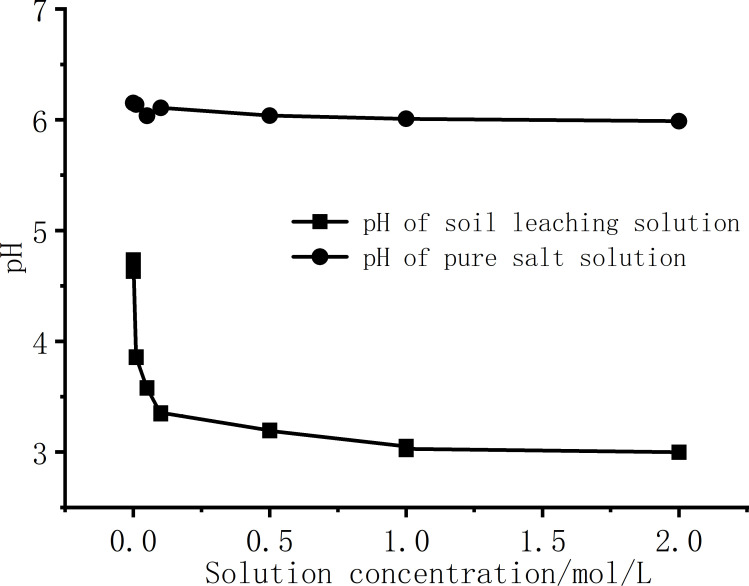
Relationship curve between solution pH value and solution concentration.


In this test, the pH value of the solution is measured by a Mettler Toledo pH meter. Fig 9 presents the pH test results of soil leachate under different NaCl solution concentrations. Because CO_2_ in the air dissolves in water and ionizes as shown in formula (3), the pH of distilled water is slightly acidic at about 6.15, which is consistent with the experimental results of Yilmaz et al., [35]. Wang et al., [36] believe that the change of salt solution concentration does not affect the pH value of pure salt solution, and the electrode of the pH measuring instrument is not be affected by the concentration of salt solution, then the pH value change of soil leachate is the result of water-soil-electrolyte system interaction. Therefore, as shown in Fig 9, the pH value of the pure salt solution remains at about 6.15, while the pH value of the soil leachate decreases with the increase of the solution concentration until it stabilizes at about 3.0. This is due to the substitution reaction of ions in the crystalline interlayer of clay minerals, Na^+^ replaces Ca^2+^, Mg^2+^, Al^3+^ and other ions in the crystalline interlayer structure, and these ions will undergo hydrolysis as shown in formulas (4)~(7), the higher the concentration of NaCl solution, the more high-valent ions are displaced, and the pH value of the solution continues to decrease until it tends to be stable.

$$CO_{2}+H_{2}O⇆HCO_{3}^{-}+H^{+}$$ (4)

$$Ca^{2+}⬝H_{2}O\overset{H_{2}O}{⇆}Ca{\left(OH\right)}_{2}+2H^{+}$$ (5)

$$Mg^{2+}⬝H_{2}O\overset{H_{2}O}{⇆}Mg{\left(OH\right)}_{2}+2H^{+}$$ (6)

$$Al^{3+}⬝H_{2}O\overset{H_{2}O}{⇆}AlHO_{2}⬝H_{2}O+3H^{+}$$ (7)

At the microscopic level, as shown in Table 2, the clay minerals in expansive soil are primarily composed of mixed-layer illite-smectite, which belongs to phyllosilicate minerals. pH testing of the solution has already indicated that interactions between pure water, salt solutions, and the soil lead to the solution becoming acidic. In an acidic environment, the surfaces and edges of clay particles generally carry negative charges. As the concentration of the salt solution increases, the thickness of the DDL on the particle surfaces gradually decreases, leading to a reduction in the repulsive forces between particles and an increase in attractive forces. The clay particles predominantly exhibit F-F contact. This results in increased cohesion between particles, making the overall structure of the expansive soil more stable. However, this tight arrangement may reduce the swelling capacity of the expansive soil. Consequently, the clay particles in the Ningming expansive soil tend to flocculate through face-to-face contact, forming aggregates. Finally, the trend in soil expansion rate corresponds with the trend in pH changes, indicating that the variation in expansion rate is consistent with the changes in the interaction patterns between soil particles.

## Expansion rate model

### Relationship between expansion rate and time

In the current research on the relationship between expansion rate and time curve, the main parameter variables are the changes of initial moisture content, initial dry density and overburden load [37,38], and the curve fitting is mainly based on the functional fitting of these parameters with the corresponding expansion rate and swelling force test results, and there is a lack of research on the influence of salt solution. In this paper, these fitting formulas are introduced to fit and compare the expansion rate and time relationship curves under the condition of different concentrations of NaCl solutions, and the fitting model with better fitting effect is selected [37,38]. The relevant fitting formulas are selected as follows (8):


$$\delta_{t}=\frac{at}{t+b}$$ (8)

Where a, b are constants, which are related to the soil material and the initial state.

**Fig 10:**
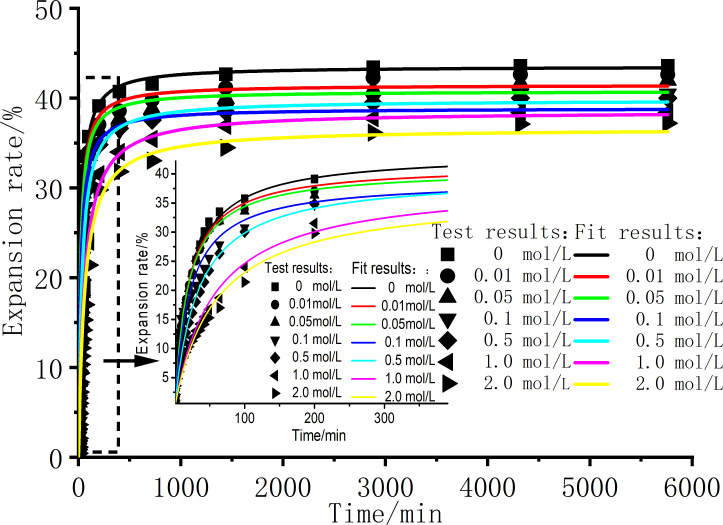
Fitting of sample expansion rate-time curves under different concentrations of solutions.

In order to verify the fitting effect of different models, the expansion rate curves of pure water solution and c = 2.0 mol/L NaCl solution were selected for the fitting of the models. Fig 10 shows the fitted relationship curves between expansion rate and time for bentonite under different NaCl solution concentrations using formula (8), and the values of the corresponding parameters *a* and *b* are shown in Table 4. It can be seen from Fig 10 that the curve fitting effect is good, and the three swelling stages of the bentonite swelling process can be well fitted. The slow swelling stage of the sample under the high concentration condition of solution c = 2.0 mol/L has a slight deviation compared with other concentrations, which may be caused by the osmotic effect existing under the condition of high concentration solution affecting the swelling of bentonite [18]. The curve fitting R^2^ is greater than 0.98, and the R^2^ is basically above 0.99, so the fitting effect of formula (8) can meet the needs of engineering applications.

**Table 4:** Results of parameters a, b for curve fitting.

Solution concentration/mol/L	a	b	R^2^
0	43.53	21.39	1.00
0.01	41.48	18.58	1.00
0.05	40.79	18.58	1.00
0.1	38.88	21.16	0.99
0.5	39.80	33.37	0.99
1.0	38.54	56.46	1.00
2.0	36.63	58.92	0.98

### Model establishment and simulation verification

#### Model establishment.

**Fig 11:**
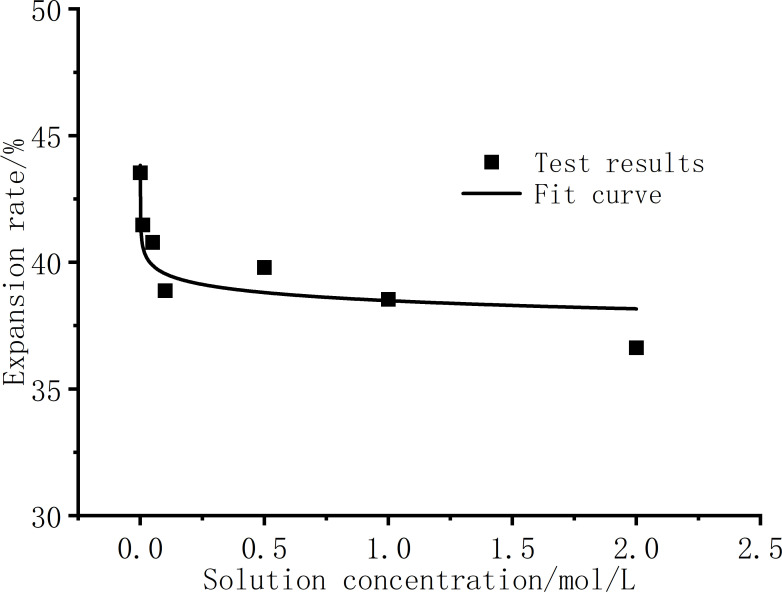
Curve fitting of parameter a.

**Fig 12:**
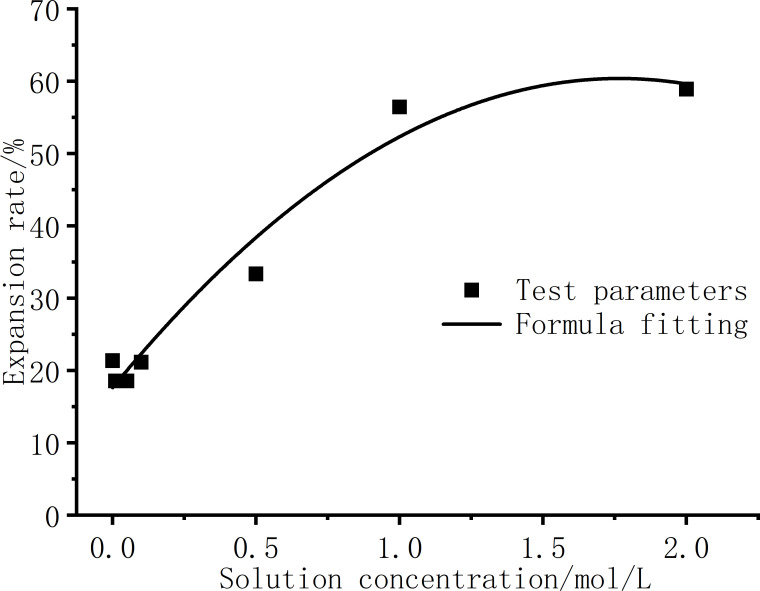
Curve fitting of parameter b.

In order to better apply the swelling curve of bentonite under the influence of NaCl solution to engineering practice and numerical simulation, it is necessary to establish a functional model related to the expansion rate and solution concentration. Table 4 shows the parameters *a* and *b* fitted by formula (8) under different concentrations of solutions, and establishes a related function model by summarizing the variation of parameters *a* and *b* with the concentration of the solution. Figs 11 and 12 respectively show the curves of parameters *a* and *b* under different solution concentrations, and perform function fitting on the curves of parameters *a* and *b* respectively, and the fitting formulas obtained according to the characteristics of the curves are shown as formulas (9) and (10). The curve fitting degree R^2^ is greater than 0.93 and 0.95 respectively, and the magnitude of parameters *a* and *b* at any solution concentration can be obtained according to formulas (9) and (10).


$$a=a_{1}\ln c+a_{2}$$ (9)

Where *c* is solution concentration, and the material parameters are *a*_1_ = -0.46 and *a*_2_ = 38.49 respectively.


$$b=b_{1}c^{2}+b_{2}c+b_{3}$$ (10)

Where *c* is solution concentration, and the material parameters are *b*_1_ = -13.70 and *b*_2_ = 48.40 respectively.

By substituting formulas (9) and (10) and the corresponding parameter sizes into Equation (8), a curve model of the expansion rate and time relationship with the solution concentration variable can be established, as shown in (11) below:


$$\delta_{t}=\frac{\left(-0.46\ln c+38.49\right)t}{t-13.7c^{2}+48.4c+17.63}$$ (11)

Where c is solution concentration, and *t* is the corresponding time in the test process.

#### Model validation.

**Fig 13:**
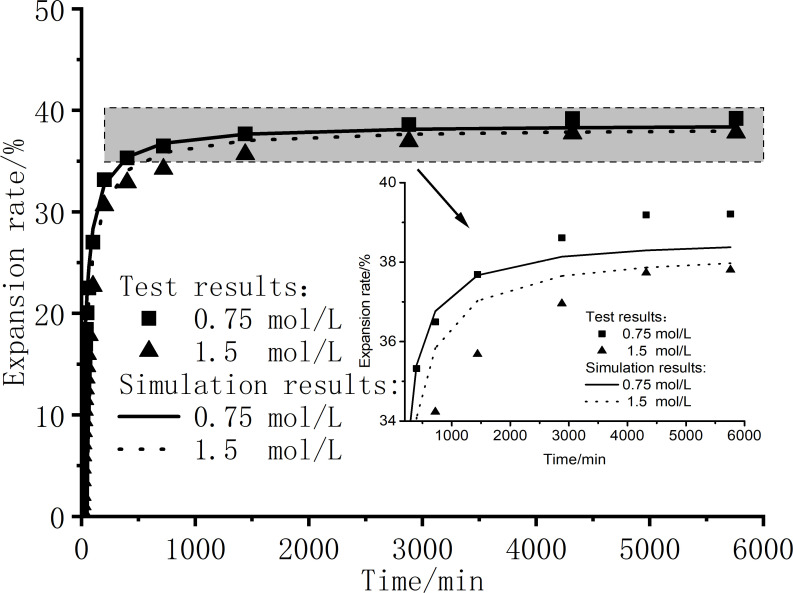
Simulation verification of expansion rate curves of different concentrations of solution.

According to the parameters obtained by fitting the above seven concentrations of NaCl solution and the established model such as Formula (11), the expansion rate and time relationship curves of bentonite with 0.75 mol/L and 1.5 mol/L salt solution other than the seven solution concentrations were simulated, and the simulation was compared with the actual experimental results. As shown in Fig 13, the deviation between the model prediction and the experimental results is very small, and the fitting degree R^2^ is greater than 0.98, which has high accuracy and consistency. As shown in Fig 13, the deviation between the model prediction results and the experimental results is very small, and the fitting degree R^2^ is greater than 0.98, which has high accuracy and consistency. It shows that the prediction model established in this paper can simulate the swelling law of Ningming bentonite under different concentrations of NaCl solution; the model is characterized by fewer parameters and high prediction accuracy; and the model has certain reference significance for the simulation of swelling of other bentonites in salt solution.

## Conclusions

When the solution concentration is below 0.5 mol/L, the swelling rate of Ningming bentonite decreases sharply. As the solution concentration continues to increase, the decline in swelling rate becomes more gradual. XRD results indicate that the interlayer spacing of montmorillonite remains unchanged, and the trend of swelling rate variation aligns with the variation trend of the double layer thickness.

With the increase in solution concentration, the MIP results at the microscopic level show that the dual-peak structure of the soil gradually evolves into a single-peak structure, with the pore size distribution predominantly consisting of medium and small pores. Corresponding SEM results reveal that clay particles transition to face-to-face contact, resulting in a more compact and uniform arrangement. Macroscopically, these changes manifest as a reduction in soil swelling. Therefore, the swelling deformation of Ningming bentonite is primarily the result of the combined effects of the diffuse double layer and particle contact modes.

By summarizing the functional relationships of relevant parameters, a swelling deformation model was developed to predict the behavior of soil under different concentrations of NaCl solution. The simulation results of the model achieved R^2^ values exceeding 0.98, demonstrating high accuracy and consistency. This model can be effectively applied to practical engineering and numerical simulation scenarios.
